# Questions Given to Applicants for Certificates to Practice Dentistry in Maryland

**Published:** 1898-04

**Authors:** 


### QUESTIONS GIVEN TO APPLICANTS FOR CERTIFICATES TO PRACTICE DENTISTRY IN MARYLAND.

Copy of questions given by the Maryland State Board of Dental Examiners, in the University of Maryland Dental Department. Thursday, November 11, 1897, to applicants for certificates to practice dentistry in the State.

### BACTERIOLOGY.

1. What is the general name of micro-organism of most importance in causing disease?
2. Can you give a further classification of bacteria?
3. How are they distinguished one from other?
4. Give some of the principal diseases caused by the bacteria.
5. What are the names of the bacteria supposed to be especially concerned in causing suppuration?
6. What is a disenfectant?
7. What is an antiseptic?
8. How are bacteria generally propagated?

### CHEMISTRY.

1. How many elements are their in nature?
2. Are they all of the same importance?
3. Give the names of some which are of great and general interest?
4. Why are these elements said to be of great and general importance?
5. Give the names of some of the less important elements?
6. Are not silver and gold platinum amongst the most important elements?
7. Why?
8. Is there any other class of elements?
9. How are these elements divided as to their physical properties?

10. Give the name of the cases?

11. Which of them is present on the earth in the largest quantity?

12. How much of the weight of the earth is made up of oxygen?

13. When was oxygen discovered, and by whom?

PATHOLOGY, THERAPEUTICS AND MATERIA MEDICA.

1. What is Pathology?
2. What is Therapeutics?
3. What is Materia Medica?
4. Describe Inflammation?
5. What are the terminations of Inflammation?
6. Name an instances where one or more of the symptoms of Inflammation may be absent?
7. What is pus?
8. Give the stages of Inflammation from its inception to the formation of pus?
9. What is Granulation?
10. What is Fever?
11. What is an Ulcer?
12. Describe caries of the Teeth?
13. Describe caries of the Bone?
14. Write a prescription containing three or more ingredients.
15. How much arsenic should be applied for distruction of dental pulp?
16. Dose of Morphia and the Antidote?
17. What is a Hæmostatic?
18. Name several?
19. What is an Anodyne?
20. Name several?
21. What is an Anaisthetic?
22. How do they affect the system?
23. What means should be used if dangerous symptoms occur?

OPERATIVE DENTISTRY.

1. Give your method of preserving the incisors when affected by caries at the age of twelve years, and name the materials you would use.

2. Give your method of treating six year molars when the nerve is slightly exposed at the age of fifteen years, all the other teeth being good.

3. Give your method of treating when the nerve is nearly exposed in the anterior teeth.

4. What is your method of capping exposed nerves, under what circumstances would you cap and what materials would you use.

5. Name drug used in destroying nerves of teeth and how applied.

6. At what period of life can six year molars be extracted with least injury to contiguous parts.

7. Describe the action of arsenious acid on the dental pulp.

8. Describe cataphoresis and name pathological conditions for which it is employed.

9. What is Epulis, give its treatment.

10. What are Alloys and what is Amalgam.

11. After devitalizing the nerve, describe subsequent treatment.

12. What is the diagnostic sign of hypersensitive dentine.

13. In respect to the properties of cohesive and non-cohesive gold, what is the distinction between them.

14. Name the different materials used for filling teeth and how they should be introduced.

15. Give full details of the operation of twelve year molars when the nerve is exposed on the posterior aproximal surface at the margin of the gum.

16. When the nerve is exposed and the teeth healthy, what is the method of treatment.

17. How do you preserve teeth, when there is much acid in the mouth, and the teeth are very much affected by it around the gum, what is your treatment.

18. What is temperament, of what use is a knowledge of it to a dentist.

PHYSIOLOGY.

1. What are the primary stages of digestion in which the mouth is concerned?

2. What are the functions of the peridental membrane.

3. What changes take place in the blood as it passes through the lungs?

4. What is meant by residual air? tidal air?

5. What proportion of the body's weight does the blood compose?

6. What is reflex action and give examples.

ANATOMY.

1. Name the bones of cranium.

2. What cavity does the superior maxillary contain.

3. Name the organs of Insalivation.

4. What are the principle muscles of Mastication?

5. Describe the ramus of the inferior Maxilla.

6. What are the branches of the fifth nerve supplying the teeth.

---

